# *ParaHox *gene expression in larval and postlarval development of the polychaete *Nereis virens *(Annelida, Lophotrochozoa)

**DOI:** 10.1186/1471-213X-8-61

**Published:** 2008-05-29

**Authors:** Milana A Kulakova, Charles E Cook, Tatiana F Andreeva

**Affiliations:** 1 Laboratory of Experimental Embryology, Biological Institute of State University of St. Petersburg, Russia; 2 Museum of Zoology, University of Cambridge, Downing St, Cambridge CB2 3EJ, UK

## Abstract

**Background:**

Transcription factors that encode ANTP-class homeobox genes play crucial roles in determining the body plan organization and specification of different organs and tissues in bilaterian animals. The three-gene *ParaHox *family descends from an ancestral gene cluster that existed before the evolution of the Bilateria. All three *ParaHox *genes are reported from deuterostomes and lophotrochozoans, but not to date from any ecdysozoan taxa, and there is evidence that the *ParaHox *genes, like the related *Hox *genes, were ancestrally a single chromosomal cluster. However, unlike the *Hox *genes, there is as yet no strong evidence that the *ParaHox *genes are expressed in spatial and temporal order during embryogenesis.

**Results:**

We isolated fragments of the three *Nereis virens ParaHox *genes, then used these as probes for whole-mount in situ hybridization in larval and postlarval worms. In *Nereis virens *the *ParaHox *genes participate in antero-posterior patterning of ectodermal and endodermal regions of the digestive tract and are expressed in some cells in the segment ganglia. The expression of these genes occurs in larval development in accordance with the position of these cells along the main body axis and in postlarval development in accordance with the position of cells in ganglia along the antero-posterior axis of each segment. In none of these tissues does expression of the three *ParaHox *genes follow the rule of temporal collinearity.

**Conclusion:**

In *Nereis virens *the *ParaHox *genes are expressed during antero-posterior patterning of the digestive system (ectodermal foregut and hindgut, and endodermal midgut) of *Nereis virens*. These genes are also expressed during axial specification of ventral neuroectodermal cell domains, where the expression domains of each gene are re-iterated in each neuromere except for the first parapodial segment. These expression domains are probably predetermined and may be directed on the antero-posterior axis by the *Hox *genes, whose expression starts much earlier during embryogenesis. Our results support the hypothesis that the *ParaHox *genes are involved in antero-posterior patterning of the developing embryo, but they do not support the notion that these genes function only in the patterning of endodermal tissues.

## Background

Transcription factors that encode ANTP-class homeobox genes; *NK*, *Rhox*, *Irx*, and in particular the *Hox *and *ParaHox *genes, play crucial roles in determining body plan organization and specification of different organs and tissues of bilaterian animals [1-7]. The *Hox *and *ParaHox *genes are believed to descend from a cluster of two to four genes that duplicated before the divergence of the Cnidaria and the Bilateria [5,8-13]: both sets of genes are ancestrally clustered on the genome, and individual genes within one cluster are paralogous with genes in the other cluster (but see [14] for a dissenting hypothesis).

Within the Bilateria *Hox *genes function to determine vectorial regionalization of the body along the antero-posterior axis and to specify body parts within this regionalization [1,2,4,15-18]. The *Hox *genes are also characterized by a high degree of structural and functional conservation: they are often (and ancestrally) found as a single chromosomal cluster, and are expressed in canonical spatial and temporal modes during embryogenesis.

The *ParaHox *genes are also believed to have originated as an organized chromosomal cluster, but the evidence for spatially and temporally collinear expression in these genes is not as strong, partially due to lack of data [8,12,19,20]. The three *ParaHox *genes; *Gsx, Xlox*, and *Cdx*, were first described as a paralogs to the *Hox *cluster in amphioxus (*Branchiostoma floridae*) by Brooke et al. [8]. A full complement of *ParaHox *genes has been shown for a number of other deuterostomes: the sea urchin *Strongylocentrotus purpuratus *[21], the hemichordate *Ptychodera flava *[22], the ascidian *Ciona intestinalis *[23], and a number of mammals, as well as for some lophotrochozoans; the sipunculids *Phascolion strombus *and *Nephasoma minuta *[23], the polychaete annelid *Capitella *sp. I [20], the clitellate annelid *Perionyx excavatus *[24], and the chiton *Nuttallochiton mirandus *[25]. However the full set of *ParaHox *genes has not yet been found in any ecdysozoan taxon, including two taxa for which complete genomes are available: the fruit fly *Drosophila melanogaster *has no *Xlox *ortholog [26,27] while the nematode *Caenorhabitis elegans *has neither *Gsh *nor *Xlox *orthologs [28].

Of the organisms studied to date the linkage has been shown only in amphioxus, mouse, and human. The *ParaHox *genes are not linked in teleost fishes [7,8], the ascidian *C. intestinalis*, or the sea urchin *S. purpuratus *[21,23].

Brooke et al. [8] reported collinear expression of the *ParaHox *genes in anterior, middle, and posterior tissues of amphioxus, and suggested that these genes may be responsible for axial patterning in the digestive tract. This pattern may be ancestral for the *ParaHox *cluster [4,8,10]. Collinear expression of two *ParaHox *genes in the digestive tract has been described for other deuterostomes: mouse and human [29,30], ascidians [31-34], and sea urchins [21]. In all cases *Xlox *(= *Pdx*) and *Cdx *expression domains were found in the central and posterior parts of the gut, respectively. The anterior *ParaHox *gene, however, is not expressed in the anterior of the gut, but rather in the brains of these deuterostomes. Holland [10] suggested that *Gsx *may have lost its function in the anterior gut as a result of changes in the patterning mechanism of the position of the mouth in deuterostomes. If this is the case then all three genes may still participate in regionalization of the gut in the protostomes (Lophotrochozoa and Ecdysozoa) [4,10].

Fröbius and Seaver [20] looked at expression of the *ParaHox *genes in a lophotrochozoan, the polychaete annelid *Capitella *sp. I, and found that *Gsx *is not expressed in the gut. This result does not support Holland's hypothesis that *ParaHox *genes are involved in gut regionalization in protostomes. However, *Capitella *sp. I is a single taxon, not basal within the annelids, and may have lost *Gsx *function independently of the deuterostomes.

In order to better understand the function of the *ParaHox *genes in the Lophotrochozoa we examined their expression in the polychaete annelid *Nereis virens*. Within the annelids *Capitella *sp. I and *N. virens *are evolutionarily remote [35,36] so *N. virens *represents an opportunity to understand, or at least make hypotheses, regarding conservation of *ParaHox *expression among the polychaetes. In addition, errant nereids are believed to retain many ancestral characteristics of the annelids, including life history, developmental and genome characteristics, homonomy of segmentation, a simple reproductive system, gradual metamorphosis, absence of highly specialized larval structures, and high fecundity [37-40], so *ParaHox *expression in *N. virens *may be more likely to represent the ancestral state.

We have cloned the full complement of *ParaHox *genes from the polychaete *Nereis virens*: *Nvi-Gsh, Nvi-Xlox*, and *Nvi-Cad*, and report larval and postlarval expression patterns for these genes in this study.

## Results

### *Nereis virens *life history

The main stages of *Nereis virens *development are presented in Table 1. The trochophore (early, middle, and late) is an unsegmented spherical larva comprising two hemispheres, the episphere and the hyposphere, whose border is marked by a belt of ciliated cells, the prototroch. The metatrochophore (early, middle, and late) larva has signs of external segmentation. The trochophore and metatrochophore are non-feeding swimming planktonic larvae. The late metatrochophore falls to the sea floor where it transforms into a benthic larva, the nectochaete, and starts feeding. The nectochaete has parapodia that it uses for crawling. During the nectochaete state a growth zone, the prepygidium, forms near the posterior of the larva and the animal then transforms into a juvenile worm. During the postlarval period of development many (about 200) postlarval segments are formed from this growth zone. Figures 7 and 8 show cartoons of these stages. More detailed descriptions of early stages of *N. virens *development can also be found in Kulakova et al. [18]. Very similar development has also been described for *Nereis limbata *[41], (now *Neanthes*, cit. by Nielsen [42]), and *Platynereis dumerilii *[43].

**Table 1:** Timing of developmental stages in the larvae of *Nereis *virens (at 10.5°C)

Stage name and time boundaries (hours after fertilization)	Brief description of main features
ET, early trochophore, 44–62	trochoblasts ciliated; stomodaeum not formed
MT, middle trochophore, 63–85	larva perfectly spherical; stomodaeum fully formed; stomodaeum lies close to the further anal region; somatic plate weakly developed
LT, late trochophore 86–105	hyposphere slightly elongated, at posterior ciliated telotroch is formed; stomodaeum and further anal region lie more widely apart from each other; chaetal sacs become morphologically apparent
EM, early metatrochophore 106–122	larva starts to show external metamery; mesotrochs starts to form in posterior part of each segment; chaetae begin to develop in two anterior pairs of chaetal sacs, but do not protrude from the larval body yet
MM, middle metatrochophore 123–152	chaetae of the two anterior pairs of chaetal sacs protrude from the larval body, chaetae of the third chaetal sac start to form, segmental boundaries become distinct
LM, late metatrochophore 153–180	larval body gradually elongates; parapodial anlagen and pygidial (anal) lobe start to form
N, nectochaete 181–390	functional parapodia; distinct head with some head appendages (two antennae and two peristomial cirri); digestive tube completely form and larvae start to eat
juvenile worm 16–17 days of development	fourth trunk segment (first postlarval) begins to form

### Expression of *N. virens ParaHox *genes in the prostomium

The prostomium of polychaetes is derived from the episphere of the trochophore. The cells of the episphere form from the first quartet of micromeres and include the head ectoderm, the cerebral ganglion, the larval and definitive eyes, the antennae, the palps, and other sensory organs of the head [41,43-45]. We have observed the expression of two *Nvi-ParaHox *genes, *Nvi-Gsh *and *Nvi-Xlox*, in the prostomium of *N. virens*.

**Figure 1:**
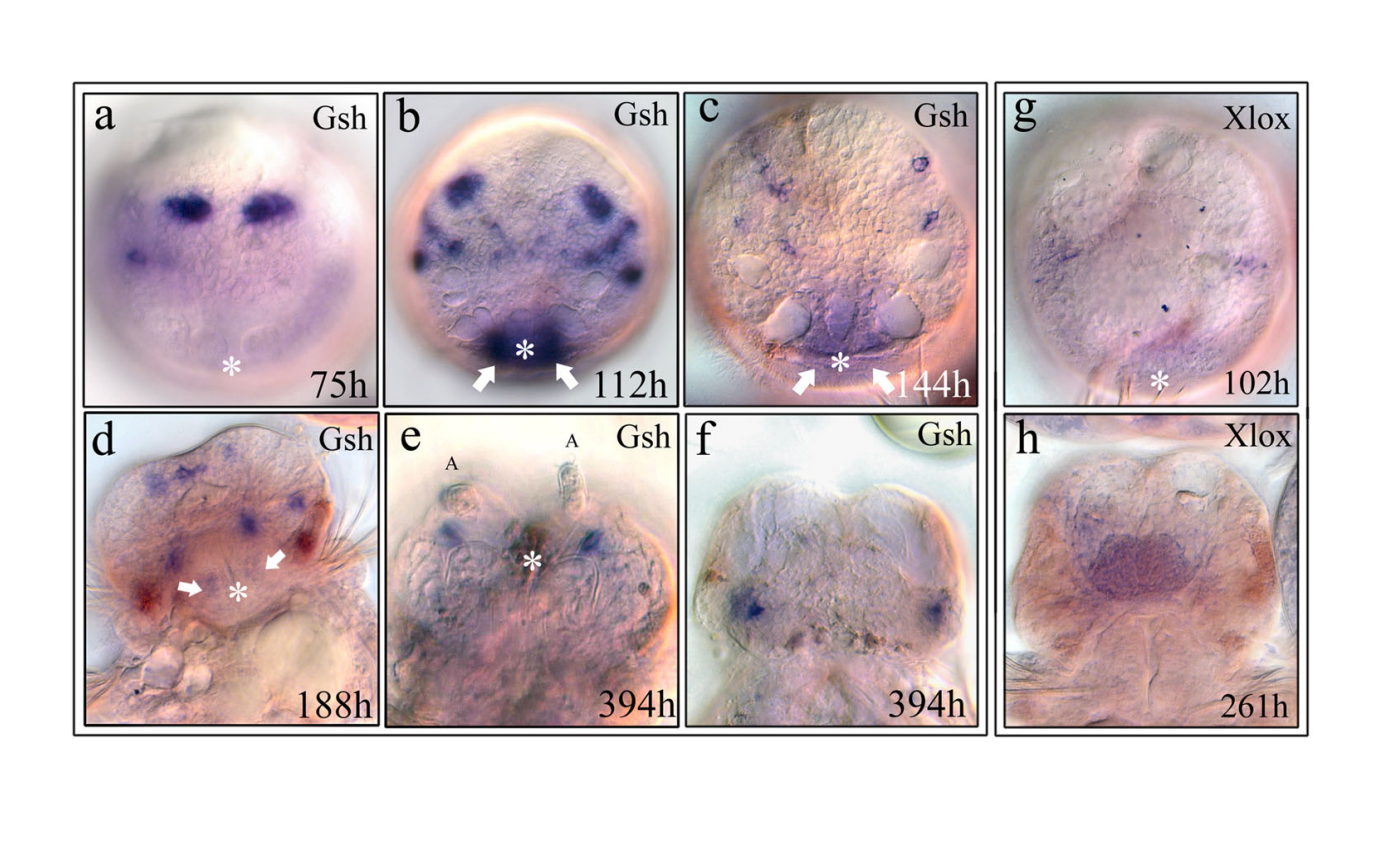
**Expression of *Nvi-ParaHox *genes in the prostomium**. *White asterisks *mark the site of the stomodeum, which is under the prototroch in the hyposphere. **(a-c) **Embryos are viewed from the animal pole. *Nvi-Gsh *is expressed in early stages in bilaterally symmetrical cells of the episphere. *White arrows *mark *Nvi-Gsh *expression in the stomodeum. **(d-f) **During the nectochaete stages *Nvi-Gsh *is expressed in a small group of cells at the ventral (**d, e**) and the dorsal (**f**) sides of the prostomium. **(g, h) **Expression of *Nvi-Xlox*. **(g) **View from the animal pole. Early expression is very weak and occurs transiently in the lateral areas of the episphere. **(h) **At the nectochaete stage *Nvi-Xlox *is expressed in a pair of lobes in the cerebral ganglion.

*Nvi-Gsh *expression begins in cells of the episphere during the middle trochophore stage when a pair of intensive, bilateral expression domains appears in the dorso-medial episphere (Fig. 1a). Later, during the early metatrochophore stage the expression pattern is more complex. Multiple bilaterally symmetrical dorso-lateral domains occur in much of the episphere (Fig. 1b). The intensity of *Nvi-Gsh *expression substantially diminishes at later stages of larval development, but is maintained in a small number of cells in a bilaterally symmetrical pattern (Fig. 1c, d). Expression of *Nvi-Gsh *in juvenile worms of 4–6 segments occurs in large cells on the ventral side of the head (Fig. 1e) and in cells at the dorsal part of the head approximately in the position of the adult eyes (Fig. 1f). There is no detectable expression of *Gsh *in larger juvenile worms.

The first detectable expression of *Nvi-Xlox *is at the late trochophore stage when a pair of weak and transitory domains occurs in the lateral part of the episphere. These symmetrical zones do not coincide with the *Gsh *expression domains (Fig. 1g). Much later, at the nectochaete stage, *Nvi-Xlox *expression resumes in a pair of lobes in the brain (Fig. 1h, Fig. 4i). This disappears at the juvenile worm stage.

### Expression of *N. virens ParaHox *genes during ventral nervous system formation

The ventral (somatic or vegetative) plate of polychaetes is derived from the 2d blastomere (the first somatoblast), which originates in the second quartet micromere. The descendants of this cell (the ectoteloblasts of Wilson [41]) intensively proliferate in the dorsal region of the embryo during gastrulation and at the trochophore stage. The lateral domains of the somatic plate become wider, move towards the ventral side and join each other between the future mouth and anus to form the ventral midline. This process leads to gradual movement of the mouth under the prototroch away from the vegetative pole that marks the position of the future anus. The vegetative pole position is stable during all of larval development. The ventral regions of the somatic plate form the neuroectoderm of the ventral neural cord and probably also the parapodial ganglia. The lateral regions of the somatic plate form the chaetae sacs, and ectoderm of the larval segments as well as the neurons of the peripheral nervous system [46]. The larval trunk of *N. virens *consists of four segments – one peristomial and three parapodial. The peristomial segment has no chaetal sacs and probably no neuromere [47], but it forms two pairs of peristomial cirri with a pair of pedal ganglia [44].

All three *N. virens ParaHox *genes are expressed in the ventral neuroectodermal cells. The activation of these genes occurs at the late trochophore/early metatrochophore stage in several symmetrical pairs of cells. The external morphological markers of segments are absent at this stage; hence the position of *ParaHox*-positive cells cannot be determined with precision. However we can compare the expression domains of *Nvi-ParaHox *genes and some *Nvi-Hox *genes. We have previously described *Nvi-Hox1*, *Nvi-Hox4*, and *Nvi-Lox5 *expression domains during early developmental stages, and these can be use as positional markers of the first, second, and third parapodial segments respectively [48].

During later developmental stages morphological markers of segments (mesotrochs) appear in the posterior of each segment. The position of *ParaHox*-positive cells can easily be compared with these markers. Note that the boundaries of segments and neuromeres in the adult worm do not coincide: the anterior boundary of the neuromeres is shifted ahead one third of a segment length [44]. This offset probably also exists for larval segments and forming neuromeres. If this is so then the anterior part of the neuromere lies exactly under the mesotroch of the preceding segment and its posterior part is at the level of its own segment mesotroch (Fig. 7).

#### Expression of *Nvi-Gsh *during formation of the ventral nervous system

**Figure 2:**
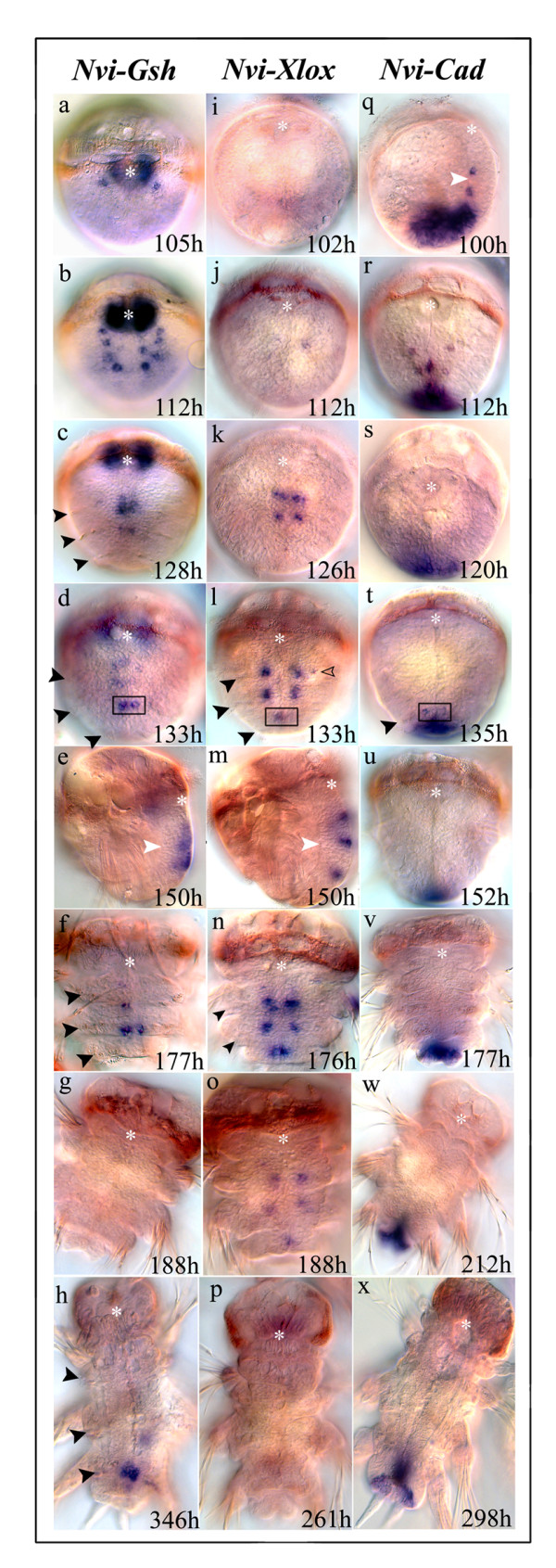
**Expression of *Nvi-ParaHox *genes during formation of the ventral neural system**. **(a – h) **Expression of *Nvi-Gsh*. **(a, b) **Early expression during the late trochophore/early metatrochophore stages. **(c, d, e) **Expression during the middle metotrochophore stage. **(e) **In some cases *Nvi-Gsh *positive cells are visible in the lateral parts of the segments. **(f, g) **At the late metotrochophore stage *Nvi-Gsh *positive cells gradually disappear from the second and then from the third parapodial segments. **(e) **In some cases *Nvi-Gsh *positive cells are visible in the lateral parts of segments (*empty arrowheads*). **(h) ***Nvi-Gsh *expression at the beginning of postlarval segmentation. **(i – p) **Expression of *Nvi-Xlox*. Expression of *Nvi-Xlox *starts later then *Nvi-Gsh *and *Nvi-Cad *(**i, j**). At the early metatrochophore stage *Nvi-Xlox *positive cells appear in the first (**j) **and later in the second parapodial segments (**k**) and during the middle metatrochophore stage *Nvi-Xlox *positive cells are visible in all parapodial segments in the central part of the forming neuromeres (**l, n**), above the mesotrochs. In some cases (**l, m**) *Nvi-Xlox *positive cells are visible in the lateral part of segments (*empty arrowheads*). At the late metotrochophore stage (**m**) short-term *Nvi-Xlox *expression occurs in the lateral cells of the peristomial segments (*white arrowheads*). During the nectochaete stage *Nvi-Xlox *expression gradually decreases (**o**) and then disappears (**p**). **(q-x) **Expression of *Nvi-Cad*. Expression of *Nvi-Cad *during the late trochophore and early metatrochophore stages (**q, r)**. This expression disappears by 120 h (**s**) but in some larvae *Nvi-Cad *positive cells can be detected during the middle metatrochophore stage (**t**) at the level of the mesotroch of the third parapodial segment. Later, *Nvi-Cad *is not expressed in neuroectodermal cells (**u-x**). During all of larval development very strong *Nvi-Cad *expression occurs in the posterior of the forming digestive system, in the forming pygidium and in the forming growth zone (see Fig. 4). View from ventral side in all cases except (**m**), which is a lateral view. *Asterisk *marks stomodeum. The mesotrochs, which are positional markers for segments, are marked by *black arrowheads*. Expression of *Nvi-Gsh*, *Nvi-Xlox*, and *Nvi-Cad *in the third parapodial segment (**d, l, t**) is indicated by *black squares*.

Expression of *Nvi-Gsh *begins during the late trochophore/early metatrochophore state, initially in one pair (Fig. 2a, 105 h) and later in several pairs (Fig. 2b, 112 h) of bilaterally situated surface neuroectodermal cells. The cells expressing *Nvi-Gsh *are in the same relative positions as those that express *Nvi-Hox1 *and *Nvi-Hox4 *– the first and second parapodial segments [18].

Middle stage metatrochophore larvae have well developed mesotrochs in the posterior part of each segment. At this stage *Nvi-Gsh *positive cells occur in the posterior part of the first and second parapodial segments parallel to or slightly posterior to the mesotroch (Fig. 2c). The *Nvi-Gsh *positive cells are thus localized in the anterior of the forming second and third neuromeres, and *Nvi-Gsh *positive zones are closer to the midline than previously as a result of rearrangement of the ventral plate cells. *Nvi-Gsh *expression in the neuromere of the first segment is no longer detected at this time (Fig. 2c). During the course of metatrochophore development the domains of *Nvi-Gsh *expression become wider in the second and third neuromere (Fig. 2d, e). Occasionally, single *Nvi-Gsh *positive cells can be found more laterally (not shown). At the late metatrochophore and early nectochaete stages the level of *Nvi-Gsh *expression gradually decreases. Still later the expression completely disappears in the second and then also in the third neuromeres (Fig. 2f, g). By the beginning of formation of the first postlarval segment *Nvi-Gsh *expression can be detected again in the posterior part of the third larval segment (Fig. 2h). This can be considered the expression in the first postlarval neuromere.

**Figure 3:**
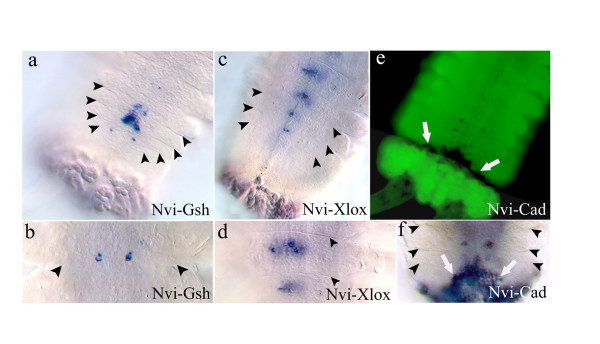
**Expression of *Nvi-ParaHox *genes during formation of postlarval neuromeres**. **(a, b) **Expression of *Nvi-Gsh*. Expression occurs in several newly formed segments **(a)**, and then disappears in the older segments until only a single pair of cells expressing the gene remains **(b)**. **(c, d) **Expression of *Nvi-Xlox*. Initial *Nvi-Xlox *expression occurs in segments that are older and more posterior than those expressing *Nvi-Gsh ***(a, c)**. *Nvi-Xlox *positive cells are localized in the anterior part of the segment below the segmental border **(d)**. **(e, f) **Expression of *Nvi-Cad*. Early *Nvi-Cad *expression occurs in newly formed segments, but this expression disappears rapidly. The worm in **(e) **was stained with TO-PRO-1. *Nvi-Cad *expression in newly formed segments can also be detected during posterior regeneration. *Nvi-Cad *positive cells are situated close to the central part of segments or a little bit to the posterior, hence in the most posterior part of the neuromere **(f)**. *Black arrowhead *marks the segmental border; *white arrowhead *marks the growth zone.

*Nvi-Gsh *is expressed in the neuroectoderm of several newly formed segments in the actively growing postlarval worm. Bilaterally symmetrical surface *Nvi-Gsh *positive cell groups are localized in the posterior zone of each segment, which is also the anterior zone of subsequent neuromeres (Fig. 3a, b). Expression begins in one cell pair in the third or fourth segment from the growth zone. These two cells are far from the midline. In the one or two immediately anterior segments the *Nvi-Gsh *expression domain is a wider multicellular zone that is closer to the midline. In older (i.e. more anterior) segments the expression is again localized in one pair of cells that is situated in the anterior part of the neuromere. In still older segments expression is absent (Fig. 3A).

#### Expression of *Nvi-Xlox *during formation of the ventral nervous system

Expression of *Nvi-Xlox *begins in the neuroectoderm at the early metatrochophore stage (Fig. 2j, 112 h). Low levels of expression can first be detected in several bilaterally symmetrical surface pairs of cells at the level of the first and second parapodial segments (Fig. 2j, k). Later *Nvi-Xlox *is also expressed in the third parapodial segment (Fig. 2l). *Nvi-Xlox *expression domains in parapodial segments become wider and stronger at middle and late metatrochophore stages. Weaker and short-term expression can also be seen in the peristomial segment at these stages (not shown). In all segments the expression domains are localized anterior to the mesotrochs and thus are in the central region of the neuromeres. In some larvae *Nvi-Xlox *positive cells also occur in the lateral regions of segments (Fig. 2l). This is analogous to expression of *Nvi-Gsh *at the same stages. Intensive expression of *Nvi-Xlox *is maintained for much longer than that of *Nvi-Gsh *(Fig. 2n, o). *Nvi-Xlox *expression gradually diminishes during the nectochaete stage (Fig. 2o), and is not detected in the neuroectoderm of the late nectochaete (Fig. 2p).

During postlarval segmentation *Nvi-Xlox *expression occurs in the forming neuromeres of newly formed segments. This surface expression can be seen in the anterior half of segments below segmental borders (Fig. 3c, d). These segments already have parapodial anlagen and cuticular furrows near nephridial pores. Expression gradually decreases in older segments that have chaetae and more developed parapodia. The youngest newly formed segments show no *Nvi-Xlox *expression.

#### Expression of *Nvi-Cad *during formation of the ventral nervous system

In the late trochophore stage pairs of cells in the ventral neuroectoderm express *Nvi-Cad *at the position where the second and third parapodial segments will be formed (Fig. 2q, 100 h). At this time the neuroectoderm consists of one or two cell layers. The duration of neuroectodermal *Nvi-Cad *expression is shorter compared to that of *Nvi-Gsh *and *Nvi-Xlox*: *Nvi-Cad *expression is not detected after 112–115 h of development (Fig. 2s, u–x). However at the middle trochophore stage weak surface expression was visible in the third segment in a single larva from among 200–300 analyzed (Fig. 2t). In these rare larvae the position of the *Nvi-Cad *expression domain can be localized because morphological markers are already formed and expression patterns of all three *Nvi-ParaHox *genes in the third forming neuromere can be compared at this stage. Using this marker we can see that *Nvi-Cad *is expressed at the level of the third parapodial segment mesotroch, *Nvi-Xlox *is expressed immediately anterior of this region, and *Nvi-Gsh *is in turn expressed in cells just anterior to the *Nvi-Xlox *expression domain (Fig. 2d, l, t). At later stages *Nvi-Cad *is not expressed in the larval neuroectoderm.

Expression of *Nvi-Cad *resumes during postlarval development in newly formed segments, where pairs of *Nvi-Cad *positive cells appear on the surface of the new segment. Expression levels are very low and the *Nvi-Cad *positive cells are better detected in TO-PRO-1 stained larvae. Bilaterally symmetrical *Nvi-Cad *positive cell groups are situated close to the center, or slightly to the posterior, of each segment, i.e. in the posterior part of the neuromere (Fig. 3e). We observed this expression pattern in only a few animals, and think it likely that this expression is transient. We found that new segments are formed faster during the regeneration of posterior parts of the growing worm. In this case the frequency of animals expressing *Nvi-Cad *is higher and transcripts can be detected more easily (Fig. 3f).

To summarize: along the zone of newly formed segments short-term and weak expression of *Nvi-Cad *occurs in a few segments, expression of *Nvi-Gsh *is stronger and occurs in older segments, and *Nvi-Xlox *expression occurs in more developed segments and lasts longer than that of *Cad *or *Gsh *(Fig. 3). Thus, the spatio-temporal dynamics of postlarval *Nvi-ParaHox *expression are very similar to those of larval stages (Fig. 2; Fig. 3). Moreover the antero-posterior positions of *Nvi-ParaHox *positive cells in each postlarval segment are also very similar to the pattern of these genes in the second and third larval segments (Fig. 2d, l, t; Fig. 3).

### Expression of *N. virens ParaHox *genes during formation of the digestive tract

#### Overview of gut formation

The digestive tract of *N. virens *is not complete until late in the nectochaete stage. Prior to this the larvae are lecithotrophic.

The foregut of polychaetes is derived from stomatoblasts [41,47]. During blastopore closure, the stomatoblasts of *Nereis virens *divide rapidly and form first the stomodaeal arch, and then the stomodaeal plate. The stomodaeal arch transforms later into the mouth, the stomodaeal plate forms the stomodaeal cavity or stomodeum by invagination. Later several sections are formed in the stomodeum: the buccal capsule, pharynx, and esophagus; the latter is connected with two large esophageal glands.

**Figure 4:**
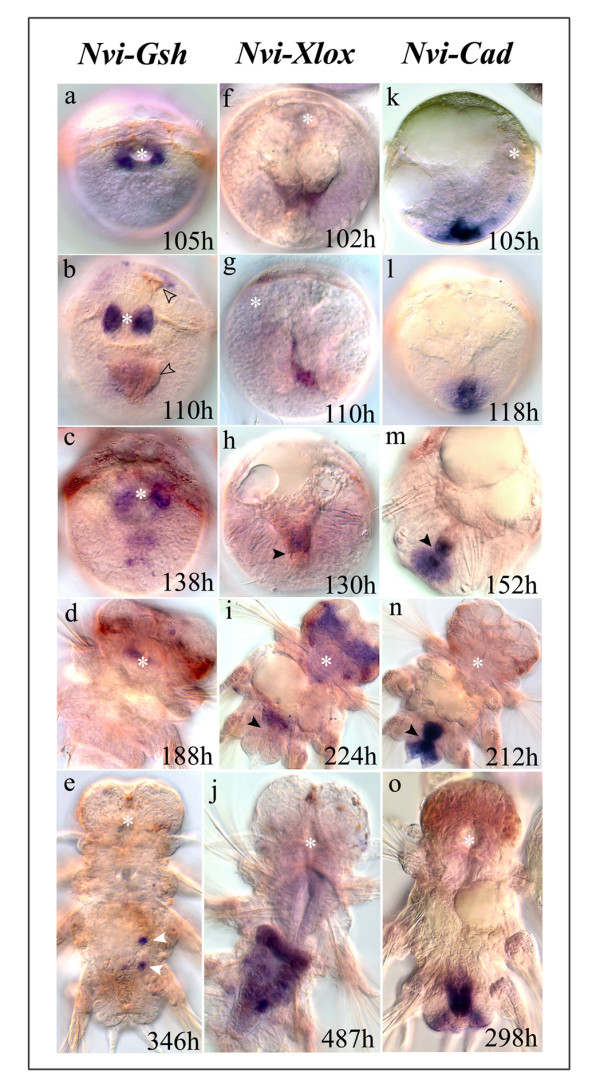
**Expression of *Nvi-ParaHox *genes during formation of the larval digestive tract**. All images are viewed from the ventral side except **h, l, m **(dorsal) and **g, k **(lateral). *The white asterisk *marks the stomadeum; *black arrowheads *mark the border between the midgut and the proctodeum; and *empty arrowheads *mark pigmented endodermal cells. **(a-e) **Expression of *Nvi-Gsh*. **(a, b) **Expression at the late trochophore/early metatrochophore stage occurs in lateral domains of the forming stomodeum. **(c, d) **Later this transcription decreases. **(e) **Transcription in the stomodeum disappears at the nectochaete stage and appears in distinct endodermal cells marked by *white arrowheads*. **(f-j) **Expression of *Nvi-Xlox*. **(f-i) **Initial expression is visible in the forming posterior endodermal domain. **(i) **At the nectochaete stage the gene is also expressed in paired lobes of the cerebral ganglion. **(j) **Later, after formation of the through-gut, *Nvi-Xlox *is expressed throughout the entire endoderm. (**k-o) **Expression of *Nvi-Cad*. **(k, l) **Initial expression occurs in the proctodaeum. **(m-o) **Later, during midgut formation, expression is also visible in the posterior region of the intestine.

The midgut of polychaetes may be divided into a stomach and an intestine proper, but in *Nereis virens *the stomach is very small or may not be present [49]. The first endodermal cells can be seen during the middle trochophore stage. These cells are formed by unequal division of very large macromeres rich with yolk. Early in midgut formation groups of endodermal cells are spread over macromeres at the vegetal pole and the episphere. These endodermal cells are easily distinguished: they are large and contain brown pigment granules (Fig. 4b). Midgut formation is complete by the end of larval development at the nectochaete stage. At this time the nectochaete starts active feeding.

The hindgut (proctodaeum) is very short and connects the anus and the midgut. Anlagen of the hindgut, pygidium, and growth zone are determined during the trochophore stage from different parts of the somatic plate. These three zones are located near each other at the vegetative pole and are surrounded by the telotroch.

#### Expression of *Nvi-Gsh *during formation of foregut and midgut

By the late trochophore stage *Nvi-Gsh *expression is activated in wide lateral domains of the forming stomodeum (Fig. 2a; Fig. 4a). The intensity of expression rises until the early metatrochophore stage, when these zones become wider (Fig. 2b; Fig. 4b). During the metatrochophore and nectochaete stages the *Nvi-Gsh *expression level gradually decreases (Fig. 2d, e; Fig. 4c, d). Expression in the stomodeum completely disappears in later nectochaete stages (Fig. 4e).

**Figure 5:**
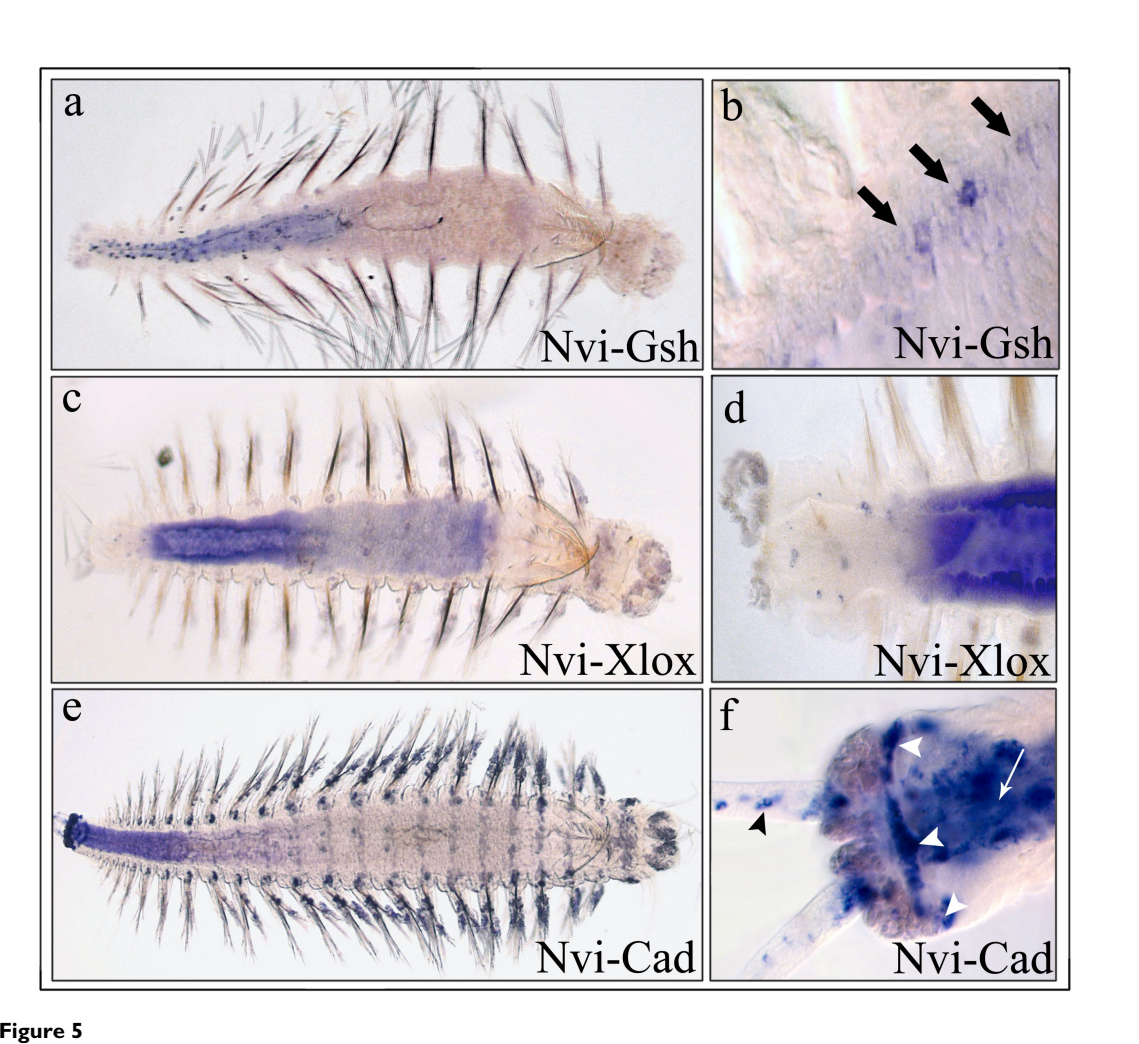
**Expression of *Nvi-ParaHox *genes during formation of the adult digestive tract**. **(a-b) **Expression of *Nvi-Gsh*. There are two cell types with *Nvi-Gsh *expression: one with very strong expression in separate, probably basal cells **(a, b)**, and an anterior to posterior double gradient of expression in the endodermal layer of the posterior half of the intestine **(a)**. That is, expression is low at the anterior of the domain, gradually increases to a peak in the center of the domain, then decreases again towards the posterior. **(c, d) **Expression of *Nvi-Xlox*. Expression in the epithelial layer occurs along the whole midgut **(c) **except at the posterior **(d)**, where distinct *Nvi-Xlox *positive cells are visible. **(e, f) **Expression of *Nvi-Cad*. There is an antero-posterior gradient of *Nvi-Cad *expression in the posterior half of the gut, with expression increasing more posteriorly **(e)**. **(f) **Three posterior domains of *Nvi-Cad *expression: midgut (*white arrow*), growth zone (*white arrowhead*), and pygidial cirri (*black arrowhead*).

Actively feeding late stage nectochaetes have some separate large *Nvi-Gsh *positive cells in the intestine (Fig. 4e). Similar large separate cells were found in different individuals containing from 4 to 25 segments in the basal layer of the intestine (Fig. 5a, b). As the midgut elongates the number of large *Nvi-Gsh *positive cells diminishes in the anterior part of the midgut and increases in the posterior. The number of these cells is distributed as a gradient along the intestine. In the growing worm *Nvi-Gsh *expression also occurs in the posterior half of the midgut in the epithelial layer of the intestine. At the ends of this domain the level of *Nvi-Gsh *expression is lower then in the central part: the zone of expression appears to be a double gradient in which the gene is expressed highly in the center while decreasing to the termini of the expression zone (Fig. 5a).

#### Expression of *Nvi-Xlox *during midgut formation

The first endodermal cells can be seen at the middle trochophore stage (80–85 h). They form small cell groups inside the vegetative and animal zones of larvae. Weak *Nvi-Xlox *expression was found only in the vegetative zone 20 hours later at the late trochophore/early metatrochophore stage (Fig. 4f, g). During the metatrochophore and nectochaete stages this gene is expressed only in the posterior part of the forming midgut (Fig. 4h, i).

As postlarval stages begin *Nvi-Xlox *expression becomes stronger throughout the midgut, and is strongest in the central and posterior part of the intestine except at the most posterior end (Fig. 4j). This pattern resembles the future postlarval one. During postlarval development in the anterior half of the intestine an antero-posterior expressional gradient forms; in the posterior half there is a double gradient in which expression is highest in the center of the domain and decreases towards each end (Fig. 5c, d). In the most posterior part of the gut separate single *Nvi-Xlox *positive cells can be detected (Fig. 5d).

### Expression of *Nvi-Cad *in posterior regions of the digestive gut and pygidium

During almost the whole of embryogenesis and larval development *Nvi-Cad *is intensively expressed in the ectodermal anlagen of the pygidial lobes, proctodaeum, and growth zone. These zones are surrounded by the telotroch. The pygidial anlage is situated near the ventral side and this domain is characterized by the earliest *Nvi-Cad *expression (40 h). Much later (100–105 h) the expression of *Nvi-Post2 *(an *Nvi-Hox *cluster posterior gene) occurs in the same zone [18]. The proctodael anlage is near the dorsal side of the embryo and does not express *Nvi-Post2*. The invagination of the proctodaeum starts during the late trochophore stage and coincides with intensive expression of *Nvi-Cad *in this zone (Fig. 4k, l). At the end of the metatrochophore stage the process of midgut formation is ongoing, the midgut joins with the proctodaeum, and *Nvi-Cad *transcription is activated in midgut cells (Fig. 4m). *Nvi-Cad *expression in the posterior part of the midgut and in the proctodaeum is maintained and intensified during the nectochaete stage (Fig. 4n).

In juvenile worms and during postlarval development *Nvi-Cad *is expressed in the same zones (Fig. 4o; Fig. 5e). In the posterior part of the gut an *Nvi-Cad *expression gradient forms with highest expression most posteriorly. This is the reverse of the gradient of *Nvi-Xlox *expression (Fig. 4j, o; Fig. 5c).

**Figure 6:**
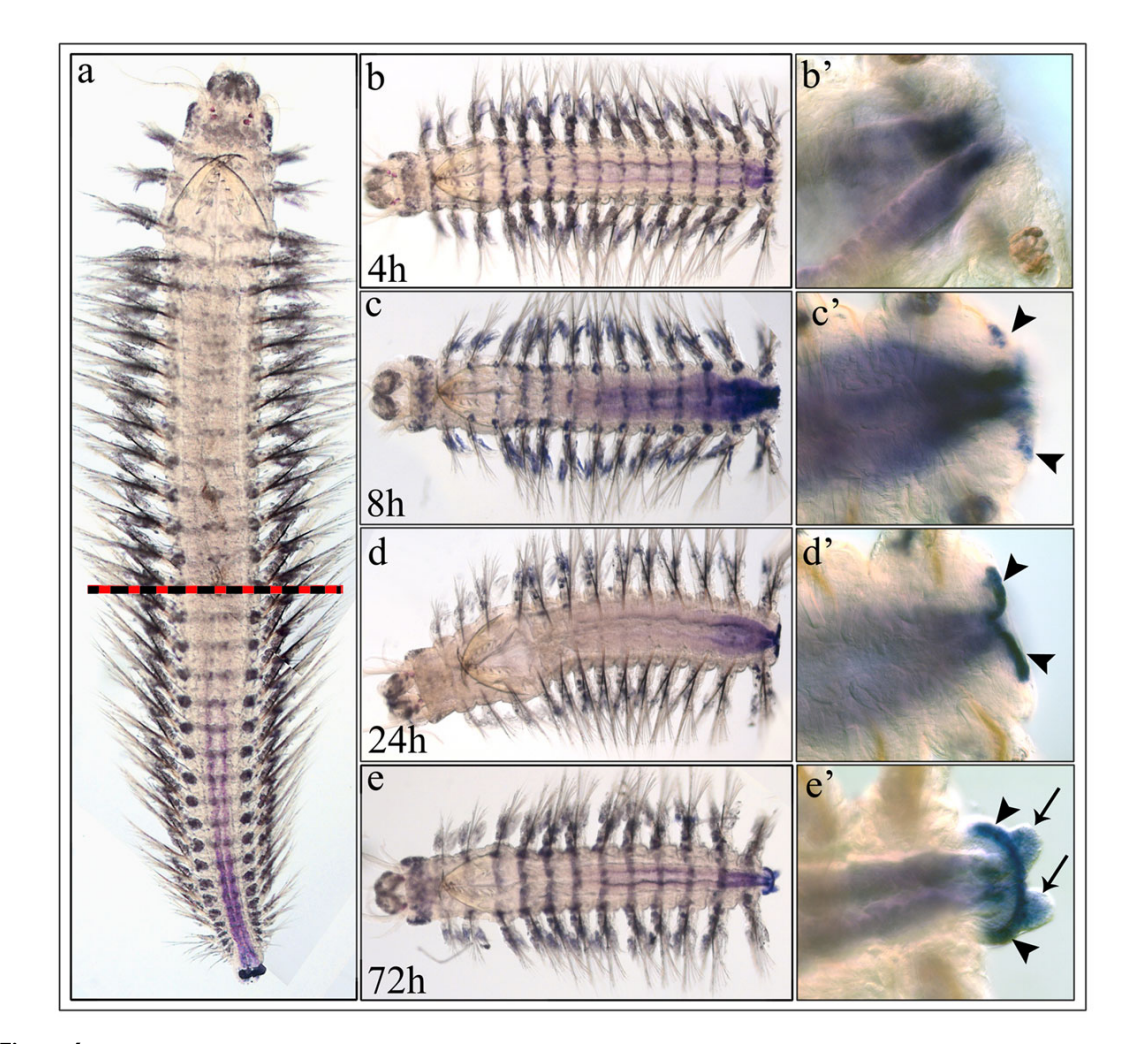
***Nvi-Cad *expression during posterior regeneration**. **(a) **Expression of *Nvi-Cad *before amputation. Striped line marks site of cut. **(b – e) ***Nvi-Cad *expression during regeneration from 4 – 72 h, as indicated in frame. **(b' – e') **Enlarged views of posterior regeneration zone in (**b – e**). *Arrowheads *mark expression in the growth zone. *Arrows *mark expression in the pygidial cirri.

During posterior regeneration upregulation of *Nvi-Cad *expression occurs in the posterior end of the remaining intestine as soon as 4 h after amputation. The amputation was done above the anterior border of the *Nvi-Cad *expression domain (Fig. 6a). In this zone the expression appears to be a short gradient (Fig. 6b, b'). Later (8 h) the length of the gradient at first increases (24 h) along almost the whole midgut (Fig. 6c, c'), and then decreases (24 h, 72 h) down to the typical domain found in uncut worms (Fig. 6d, d', e, e'). *Nvi-Cad *expression also marks the newly formed growth zone, and pygidium (Fig. 6c', d', e').

## Discussion

Holland [10] and Garcia-Fernandez [4] have suggested that the origin of the Bilateria, with three germ layers, was associated with the origin of several gene clusters in the ANTP family: the *Hox*-cluster genes participated mainly in regionalization of the neuroectoderm, the *NK*-cluster genes took part in patterning the mesodermal layers, and *ParaHox *patterned the endoderm. However, previous to our work on *N. virens*, no bilaterians are known in which all three *ParaHox *genes participate in patterning of the gut.

In deuterostomes the patterning of the digestive system is clearly different from the ancestral state since the mouth forms in a different position in deuterostome embryos, thus changes in the function of genes controlling development of the gut are not unexpected. In deuterostomes all three *ParaHox *genes are found, but *Gsx *is not, or is no longer, expressed in the anterior of the gut [10].

All three *ParaHox *genes have been found in a number of lophotrochozoan taxa [20,24,25,50], but expression of the full set has to date been described only for the polychaete *Capitella *sp. I [20]. In this species *Gsx *is also not expressed in the gut, but is, as in many other bilaterians, associated with brain development.

### Are *ParaHox *genes expressed ancestrally in the brain?

*Gsx *is expressed in the brain during development of all bilaterians studied to date, including *N. virens*. *Nvi-Gsx *is activated early, during the middle trochophore stage, in dorso-lateral regions of the head neuroectoderm. Expression of this gene is intensive, prolonged, and dynamic (Fig. 1). Maps of presumptive brain anlagen for a related species, *Platynereis dumerilii *[45] can be correlated with development in *N. virens *and allow us to locate *Nvi-Gsx *expression domains with optic areas and possibly with regions of prospective antennae. In another polychaete, *Capitella *sp. I, *CapI-Gsx *is also expressed in the head neuroectoderm, and is indeed the only expression domain for this gene in that species [20].

In *D. melanogaster *the *Gsx *homologue, *ind*, is expressed in a subset of brain neuroblasts [51]. *Ci-gsx *in the ascidian *Ciona intestinalis *was found in the sensory vesicle, which is thought to be homologous to the vertebrate forebrain and midbrain [33]. *AmphiGsx *is expressed in the cerebral vesicle in amphioxus [8]. *Gsh1 *and *Gsh2 *of the mouse take part in patterning of the telencephalon, particularly in the lateral ganglionic eminence and the olfactory bulb [52-54]. *Gsh-1 *of the mouse and *Ol-Gsh1 *of the medaka, *Oryzias latipes*, have similar expression patterns in the optic tectum, dorsal diencephalon, hypothalamus, and rostral telencephalon [55,56]. Thus *Gsx *expression has been observed in the brain of all three major branches of the Bilateria.

The *Gsx *homologue *anthox2 *in *Nematostella vectensis *(Anthozoa), which is a basal representative of Cnidaria, is expressed around the oral pole in scattered ectodermal cells that are probably neural cells [14,57]. In another diploblast, the coral *Acropora millepora*, the *Gsx *homologue *cnox-2Am *is also expressed in ectodermal (probably neural) cells of the oral region [58]. Thus in Bilateria and Cnidaria *Gsx *is expressed in neural cells, and may have an ancient role in patterning the neural system of the ancestral bilaterian [14,57,58].

We also observed that very weak and transitory *Nvi-Xlox *expression occurs in the episphere of the trochophore and in the cerebral ganglion of the *N. virens *nectochaete. These early expression domains do not correlate with any identified brain anlagen or with zones of *Nvi-Gsh *expression (Fig. 1).

In the polychaete *Capitella *sp.I *Cap*-*Xlox *expression is not detected in head neuroectoderm or any other derivatives of the brain [20]. The *Xlox *homologue of the rat, *IDX1/IPF *is expressed in several areas of the developing brain, including the cortex, ganglionic eminence, hypothalamus, and inferior colliculus [59,60]; and *Ci-IPF1*, the *Xlox *homologue in the acsidian *Ciona intestinalis*, is expressed in the larval sensory vesicle [34].

*Nvi-Cad *is not expressed in the brain of *Nereis virens *at any studied developmental stage. In *Capitella *sp.I *CapI-Cdx *expression was detected in multiple domains of the developing larvae. *CapI-Gsh *and *CapI-Cad *areco-expressed in neuroectoderm during the early stages of brain development [20].

In sum, *ParaHox *genes are expressed in the brains of all studied bilaterian animals but in none of them are all three genes expressed. Expression in the head neuroectoderm is always found for *Gsx *and is probably the ancestral characteristic for the last common bilaterian and cnidarian ancestor. The two other *ParaHox *genes are not always expressed in the head neuroectoderm: *Xlox *is expressed in a few studied species, and *Cdx *in only one, the polychaete *Capitella *sp. I; we cannot at present make any hypotheses regarding their ancestral expression patterns in the brain, and await further data.

### Expression of *ParaHox *genes in the trunk neuroectoderm

Expression of all three *ParaHox *genes in ventral, or dorsal, neuroectoderm has not been demonstrated in any studied species. Mice, humans, and probably *Xenopus *as well, have only one *ParaHox *cluster, *Gsh1, Ipf1*, (= *Pdx1*, = *Xlox*), and *Cdx2*; as well as two additional orphan *Cdx *genes (*Cdx1 *and *Cdx4*) and one orphan *Gsx *gene (*Gsh2*) [7,61]. *Gsh-1 *of the mouse (and *Ol-Gsh1 *of the medaka fish) is expressed in the hindbrain [55,56]. It has been shown that *Gsh1 *as well as *Gsh2 *take part in dorso-ventral patterning [27,62]. The *Gsx *homologue in *D. melanogaster*, *ind*, also takes part in dorsoventral specification of the nerve cord [27]. *IDX1/IPF1 *(*Xlox*) of the rat is also expressed in the hindbrain at embryonic day 15 [59].

All three mouse *Cdx *genes are expressed in the posterior of the neural tube, and their expression domains overlap considerably [63,64]. In zebrafish there is no *Cdx *expression in the progenitor cells of the hindbrain, but loss of function of *Cdx1 *and *Cdx4 *or gain of function of *Cdx4 *result in displacement of the hindbrain-spinal cord boundary (i.e. a homeotic transformation). These genes may determine axial regionalisation in the trunk neural cord [65].

In amphioxus *AmphiGsh *is not expressed in the trunk neuroectoderm, *AmphiXlox *is expressed in pigment cells, and *AmphiCdx *in the most posterior region of the neural cord [8]. *Ci-IPF1 *(*Xlox*) of *Ciona intestinalis *is expressed in the visceral ganglia, which are thought to be homologous to the vertebrate hindbrain/spinal cord region [34]. In another ascidian, *Herdmania curvata*, *Hec-Cdx *is expressed in the posterior of the neural tube [32].

All three *ParaHox *genes are found in the polychaete *Capitella *but only one gene, *Capl-Cdx*, is expressed in posterior neuroectodermal cells [20]. *Platynereis dumerilii *(a polychaete) also has a full set of *ParaHox *genes (our unpublished data for *Pdu-Xlox*; ref. [46] for *Pdu-Gsh*; and ref. [66] for *Pdu-Cad*)). *Pdu-Gsx *is expressed in a central part of the larval ventral neuroectoderm (*Pdu-nk2.2 *positive domain) in which somatic serotonergic motoneurons are identified and differentiated. *Pdu-Gsx *is also expressed at late stages of neuroectoderm formation and is associated with cell differentiation. An ordered disposition of different zones along the apicobasal axis has been shown for *P. dumerilii*: there is a zone of proliferation (more apically), a progenitor zone of postmitotic cells in which neuronal identity genes are expressed, and a zone of differentiation (more basally) [46]. *Platynereis dumerilii *and *Nereis virens *develop similarly and show identical patterns of *Hox *gene expression [18]. In *N. virens ParaHox *positive cells are situated in the zone of proliferation and in the progenitor zone (Fig. 2), which we suggest indicates that these genes take part in cell division and/or cell patterning processes. The *expression of the *ParaHox *genes *in order along the antero-posterior axis also suggests that these genes have a patterning function in *N. virens*.

### Axial pattern of *Nvi-ParaHox *gene expression in the forming neuromeres

To date, the polychaete *Nereis virens *is the only studied animal in which all the *ParaHox *genes are expressed in the ventral neural system. *Nvi-ParaHox *gene expression is so far unique in being metameric: the expression patterns of each *ParaHox *gene are found in repeating cell groups of each larval and postlarval segment, but at different times during development. Exceptions are the peristomial and the first parapodial segments of the larva, where expression of *Nvi-Cad *is not detected (Fig. 2; Fig. 3). These particular segments will be included in the head of the adult worm, and their ganglia will differ morphologically and probably functionally from the rest of the neuromeres.

### During larval development

**Figure 7:**
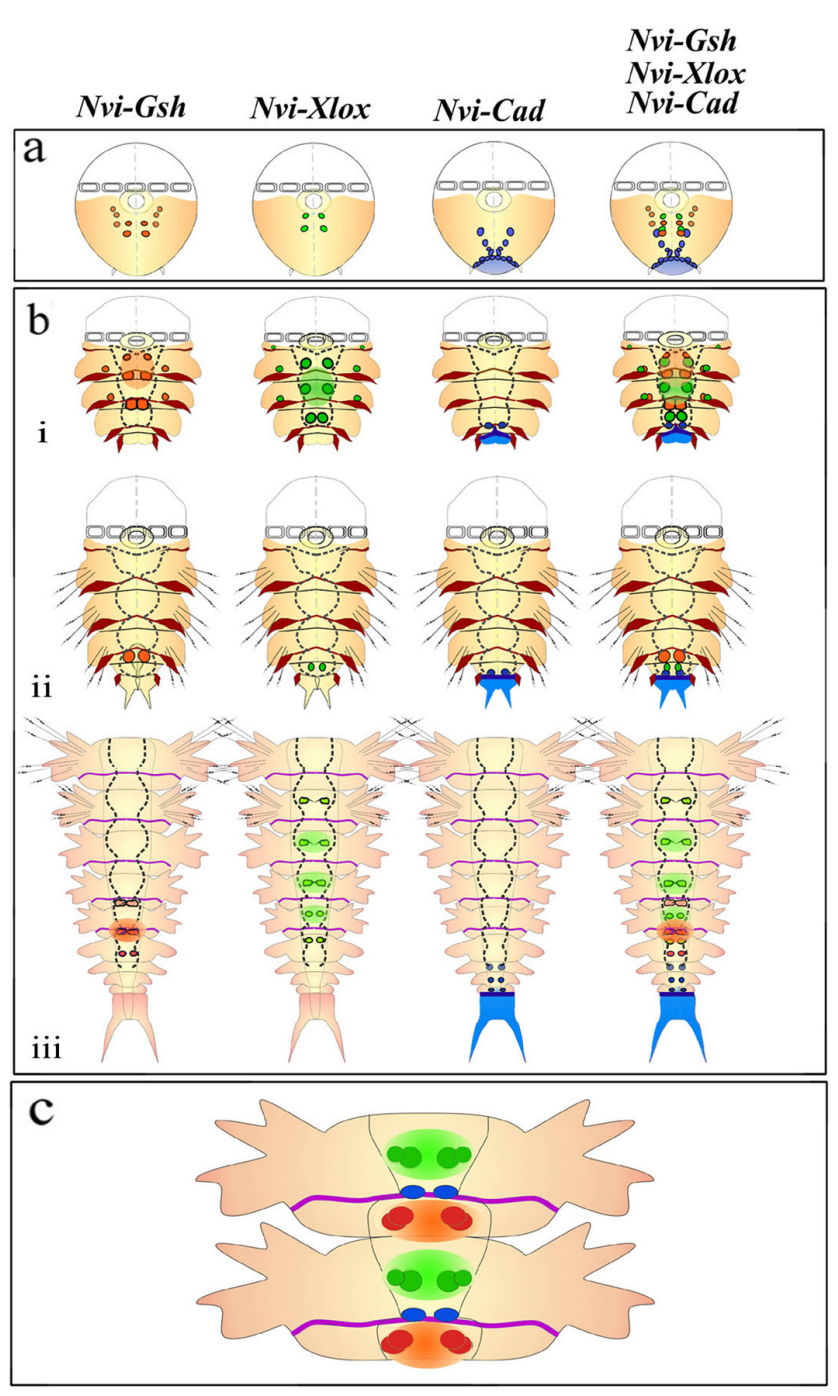
***Nvi-ParaHox *gene expression in the forming ventral nervous system**. **(a) **Early expression at late trochophore/early metatrochophore stage. At this stage there are no morphological markers of segmentation present. All three genes are expressed in a collinear order, with *Nvi-Gsh *most anterior, *Nvi-Xlox *more central, and *Nvi-Cad *most posterior. **(b) **Expression of *Nvi-ParaHox *genes at the metatrochophore **(i)**, late nectochaete/juvenile worm **(ii) **stages, and during postlarval segmentation **(iii)**. **(c) **Hypothetical scheme of distribution of *Nvi-ParaHox *positive cell descendants in well-developed segments. *The black dotted line *marks the boundaries of forming neuromeres. *Black lines ***(bi, bii) **mark the boundaries of well developed neuromeres. *Brown lines ***(bi, bii) **mark the positions of mesotrochs. The violet line **(biii, c) **marks cuticular furrows of well developed segments. *The dark blue line ***(b) **marks *Nvi-Cad *expression in the growth zone. *Nvi-Cad *expression in the pygidium is denoted with *light blue ***(a, b)**.

*N. virens ParaHox *gene expression exhibits spatial collinearity. Figure 7 shows the primary expression of the *Nvi-ParaHox *genes during ventral nerve cord formation. *Nvi-Gsh *is expressed in some cells of all the larval neuromeres; these cells are located in the most anterior part of each neuromere. Cells expressing *Nvi-Xlox *are also expressed in all neuromeres, but in their central regions, so that the anterior border of *Nvi-Gsh *expression is always anterior to the anterior border of the *Nvi-Xlox *expression domain. *Nvi-Cad *expression occurs in some cells of the second and third neuromeres and in these neuromeres is posterior to the anterior-most expression of either *Gsh *or *Xlox*. The spatially ordered distribution of *Nvi-ParaHox *expression domains (Fig. 2; Fig. 7) is thus similar to ordered *Nvi-Hox *expression in the larval ventral plate [18]. However, *Hox *expression domains include all cells of the somatic plate (ectodermal and neuroectodermal) whereas *ParaHox *genes specify small groups of particular neuroectodermal cells (precursors of motorneurons) in the central part of the neural plate [46].

By contrast, the *Nvi-ParaHox *genes do not exhibit temporal collinearity of expression. *Nvi-Cad *is activated in the neuroectoderm a little earlier than *Nvi-Gsh *or simultaneously with it, and *Nvi-Xlox *is activated later than the two other genes (Fig. 2). Interestingly, larval *Nvi-Hox *genes also lack temporal collinearity of expression: the posterior gene *Nvi-Post2 *is expressed earlier then the genes of the central group: *Nvi-Hox7, Nvi-Lox4*, and *Nvi-Lox2*, and *Nvi-Hox3 *is expressed before *Nvi-Hox1 *[18].

### During postlarval development

All *Nvi-ParaHox *genes are expressed in each segment, but in different cell groups of the forming neuromere and at different times during segment morphogenesis. In every neuromere the spatial domains of *Nvi-ParaHox *expression are shifted relative to each other and specify different groups of neurons along the antero-posterior axis in apparent accord with the linear position of the genes in the *Nvi-ParaHox *cluster [67] (Fig. 7). In this case, however, collinearity can be seen in every neuromere but not in the whole trunk.

*Nvi-Cad *demonstrates the earliest and most transient expression in the two or three youngest postlarval segments. Expression of *Nvi-Gsh *and *Nvi-Xlox *is activated in older segments, but there are more *Nvi-Xlox *expressing segments than *Nvi*-*Gsh *ones, suggesting that *Nvi-Xlox *expression is longer lasting than that of *Nvi-Gsh*. We did not observe simultaneous expression of all three *ParaHox *genes in the same postlarval segment. During postlarval development a dynamic morphogenetic system is formed that includes an active growth zone and many segments at various stages of maturity. The difference in time of activation and down regulation of different *ParaHox *genes leads to full segmental separation of their expression domains.

### Axial patterning of *ParaHox *gene expression in the digestive system

**Figure 8:**
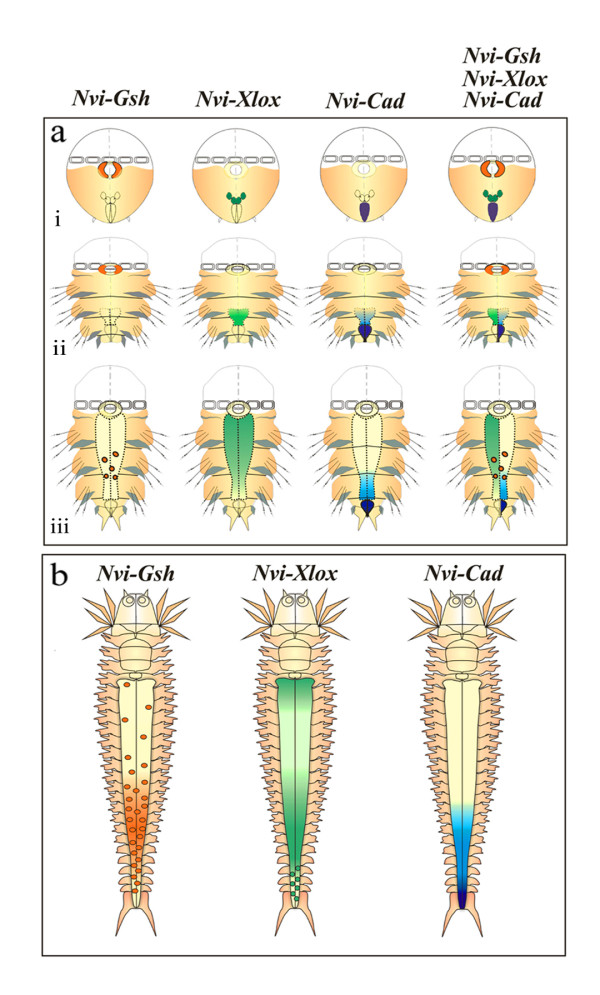
**Presumptive scheme of *Nvi-ParaHox *gene expression in the forming digestive system**. **(a) **Larval expression of *Nvi-ParaHox *gene at the trochophore (**i**), metatrochophore (**ii**), and nectochaete (**iii**) stages. *Dotted line *marks the forming midgut. **(b) **Expression patterns of *Nvi-ParaHox *genes during postlarval stages.

We show that all three *ParaHox *genes are expressed in the digestive system of *N. virens*. This is the first such report for any lophotrochozoan taxon. During larval development *Nvi-ParaHox *gene expression in the gut is also spatially collinear: *Nvi-Gsh *is expressed in the foregut, *Nvi-Xlox *in the midgut, and *Nvi-Cad *in the hindgut. As with in other cell types, however, there is no temporal collinearity. *Nvi-Cad *expression begins at 40 h, much earlier than *Nvi-Gsh *at 100 h, and initial expression of *Nvi-Xlox *and *Nvi-Gsh *is almost simultaneousl. Expression of the different *Nvi-ParaHox *genes coincides with the period during which the various digestive tract primordia form, and these do not form in anterior to posterior sequence. The late development of the midgut may be a response to the large yolk resource which is a characteristic of lecitotrophic development. The main regions of *Nvi-ParaHox *gene expression during gut formation are shown in Fig. 8.

During larval development *Nvi-Gsh *is first expressed in the foregut, where it is later downregulated and then expressed in basal cells of the midgut (Fig. 4; Fig. 8). During postlarval development the number of basal cells increases towards the posterior end of the midgut and the expression pattern looks like a gradient. We will abbreviate this type of antero-posterior gradient as **a-P**. *Nvi-Gsh *expression in the epithelial layer of the posterior domain is a two sloped gradient **p-P-p **(Fig. 5; Fig. 8). During larval development *Nvi-Xlox *marks the posterior endodermal cells of the forming midgut, and in postlarval development the expression forms an **A-a-p-P-p **pattern along the intestine (Fig. 4; Fig. 5; Fig. 8). *Nvi-Cad *is expressed in the forming proctodaeum before and after its invagination. Later this gene is also expressed in the posterior of the larval midgut and at this time a **p-P **expression gradient in the midgut starts to form. This pattern is retained during postlarval development (Fig. 4; Fig. 5; Fig. 8).

During postlarval development, then, the spatial expression pattern of *Nvi*-*ParaHox *genes in the digestive system does not substantially change. As the worm becomes longer the length of the digestive tract is also increased and along it all the established gradients are maintained (Fig. 5; Fig. 8). During this period the function of the *Nvi-ParaHox *genes may be related to the maintainence of regional specificity (i) and cell differentiation (ii) along the antero-posterior axis of the gut.

(i) The very rapid reorganization of the *Nvi-Cad *endodermal expression pattern during regeneration (Fig. 6) points to a regional patterning function of this gene. We detected upregulation of this gene in the most posterior part of the bisected worm as soon as 4 h after amputation. This is not enough time for dedifferentiation of old cell types and the differentiation of new ones: we suggest that this is evidence that new positional information is created in old differentiated cells.

(ii) It is possible that *Nvi-ParaHox *genes take part in cell differentiation. The gradient distribution of different cell types along the intestine [49] probably coincides with the gradient expressional patterns of *Nvi-ParaHox *genes (Fig. 5). For example, the number of secretory and absorptive cells in the epithelial layer gradually decreases in the posterior part of the gut [49]. This is correlated with the gradual diminishing of *Nvi-Gsh *and *Nvi-Xlox *expression and very high expression levels of *Nvi-Cad *in the posterior of the gut; the number of basal cells gradually increases in the posterior of the intestine, and is correlated with the **p-P **gradient of *Nvi-Cad *and **a-P **gradient of *Nvi-Gsh *in basal cells of the intestine (Fig. 5; Fig. 8).

These expression patterns are similar to patterns of *Pdx-1 *(*Xlox*) and *Cdx-2 *along the small intestine of the adult mouse. Fang et al. [30] showed that these genes form two opposite and overlapping expression gradients in this part of the gut, and that the transcription of some digestive enzymes is activated by *Cdx-2 *and repressed by *Pdx-1*. Thus two overlapping gradients of upstream regulators take part in functional regionalization of the small intestine [30].

During mouse embryogenesis *Pdx-1 *also takes part in the patterning of the pancreas and the anterior part of the duodenum. *Pdx-1 *inactivation results in atrophy of the pancreas and defects in the anterior part of the duodenum [68]. Inactivation of *Cdx-2 *results in homeotic transformation: stomach tissue is induced in the midgut and hindgut [69]. Moreover *Cdx2 *is essential for axial elongation in the mouse [63]. Interestingly, all *ParaHox *genes are expressed in the pancreas of the adult mouse [70].

During development in two deuterostomes; amphioxus and the sea urchin, only two *ParaHox *genes, *Xlox *and *Cdx*, take part in patterning and specification of different regions of the digestive tract [8,21]. Holland [10] suggested that this function has been lost for *Gsh *because the position of the mouth has changed in this branch of the Bilateria. Our results, showing that all three *ParaHox *genes are expressed in a lophotrochozoan, support the hypothesis that *Gsh *patterning of the gut has been lost in at least some of the deuterostomes.

Fröbius and Seaver [20] described the expression patterns of the *ParaHox *genes in the polychaete *Capitella *sp. I. They did not observe *CapI-Gsx *expression in the digestive tract, but did see brief expression of *CapI-Xlox *in the forming midgut and expression of *CapI-Cad *in the posterior part of the gut. Thus expression of *ParaHox *genes in *Capitella *and their expression in *N. virens *are very different: this is not surprising since the two species are phylogenetically distant from one another [35,36].

Errant nereids are thought to retain many ancestral polychaete characters [37-40]. If this is also true of gene expression then the involvement of all three *ParaHox *genes in the development of *N. virens *may more closely reflect the ancestral use of these genes, thus supporting the idea that the three *ParaHox *genes, like the *Hox *genes, were ancestrally involved in antero-posterior patterning during bilaterian development.

## Conclusion

In the Bilateria the role of *Hox *genes in antero-posterior axial regionalization is well established, and the ancestral function of these genes has been in patterning and specification of ectodermal and neuroectodermal anlagen. The ancestral role of the *ParaHox *genes is less well established; it has been suggested that these genes might have a parallel role in antero-posterior patterning in the endodermal layer of the developing embryo [4,10].

This hypothesis has been partially supported by studies in deuterostomes and ecdysozoans, but to date there are no taxa from either group for which all three *ParaHox *genes are expressed in developing endodermal tissues: because one or more *ParaHox *genes have been either lost or coopted for other functions. However, in the sole lophotrochozoan taxon studied to date, *Capitella *sp. I, one of three genes (*CapI-Gsh*) has no function in endodermal patterning. In aggregate, these studies show that all three *ParaHox *genes are expressed in some endodermal tissues in at least some bilaterians, but in no single taxon are all three genes expressed in endodermal or antero-posterior patterning.

In this paper we describe the expression patterns of the three *ParaHox *genes in another polychaete, *Nereis virens*, during formation of the digestive system and the ventral nerve system. We found that all three *ParaHox *genes are expressed during antero-posterior regionalization of the gut, and that these genes are transcribed along the main body axis in a collinear order that involves both ectodermal (foregut and hindgut) and endodermal (midgut) regions of the digestive tract. During ventral nerve cord formation the *ParaHox *genes are expressed during specification of some cells in the ganglia of larval and postlarval segments. The well-ordered expression of these genes occurs in *larval development *in accordance with the position of these cells along the main body axis. During *postlarval development *these genes are expressed in order in accordance with the position of cells in ganglia along the antero-posterior axis of each segment. In none of these tissues are the three *ParaHox *genes expressed following the rule of temporal collinearity.

The expression of *ParaHox *genes during antero-posterior development of the digestive system (ectodermal foregut and hindgut, and endodermal midgut) suggests that these genes are actively involved in antero-posterio specification in the *N. virens *gut. Similarly, the segmental expression of *Nvi-ParaHox *genes in each neuromere except in the first parapodial segment, suggests that these genes also take part in axial specification of ventral neuroectodermal cell domains. We hypothesize that these expression domains may be predetermined and directed on the antero-posterior axis by the *Hox *genes, whose expression starts much earlier during embryogenesis, and along the mediolateral axis by the BMP-regulatory system of the *Pax *and *NK *genes [18,46]. Our results lend support to the hypothesis that the *ParaHox *genes, like their sister *Hox *genes, are involved in antero-posterior patterning of the developing embryo, but they do not support the notion that these genes function only in the patterning of endodermal tissues. Confirmation of the function of the *ParaHox *genes awaits additional descriptive data from other taxa, as well functional studies that manipulate gene expression during development in a lophotrochozoan taxon.

## Methods

### Animals

Adult *Nereis virens *were collected at the Chupa Inlet Marine Biological Station of St. Petersburg State University, on the White Sea. Mature animals were caught with a hand net at the water surface during the spawning period (June and July). Artificial fertilization and cultivation of the embryos were carried out at 10.5°C (Dondua, 1975).

### Cloning of short homeobox-containing fragments of Nvi-ParaHox genes

We have previously described the use of degenerate primers complementary to the most conserved motifs in the homeodomain to amplify *Hox *genes from *N. virens *[18,66]. These screens, using forward primers GARCTIGARAARGARTT (1798C), CTIGARCTIGARAARGA (Fbam), YTIGARYTIGARAARGART (AN1); and reverse primers ICGRTTTTGRAACCA (Rxba), CGYTTRTTTTGRAACCA (Rend), and ATTCATICKICKRTTYTGRAACCAIATYT (AN2), produce PCR amplicons that are mixtures of different homeobox-containing fragments. We isolated individual sequences by cloning the PCR products into pBluescript SK+ at the EcoRV restriction site, then growing and sequencing individual clones. These clones included the eleven *Hox *genes that we have previously reported, and the *ParaHox *genes that we report herein. It is important to note that each primer pair produces a different suite of amplified genes, so thorough screens of this type benefit from the use of many primer pairs. Of the nine different primer combinations that we used we recovered *ParaHox *sequences from only four: *Nvi-Gsh *was obtained with the 1798C/AN2 primer pair, *Nvi-Xlox *with AN1/Rxba and AN1/AN2, and *Nvi-Cad *with AN1/AN2 and Fbam/Rend.

The short gene fragments were identified using Wu-BlastX searches against GenBank. Gene identities were confirmed by additional Blast searches after longer sequences became available. The sequences have been deposited in GenBank with accession numbers AY117546, DQ206221, and DQ366677.

### Cloning of long *Nvi-Gsh*, *Nvi-Xlox*, and *Nvi-Cad *fragments using inverse PCR and 3' RACE

We used inverse PCR (iPCR) and 3' RACE to extend the *ParaHox *gene sequences outside of the short fragments obtained using degenerate primers. We have described iPCR in detail elsewhere [71]. Briefly, *Nereis virens *DNA was digested with the following restriction endonucleases: *EcoRI, BamHI, HindIII*, and *AluI*, and the DNA fragments obtained were ligated under conditions that ensured self-ligation, resulting in circularized DNA. This DNA was then used as template for PCR. Pairs of nested primers for each small fragment of *ParaHox *gene were constructed in such a way that the ends of the amplified iPCR fragment were in the middle of an earlier known sequence. Amplified products were cloned in the T Easy vector (Promega), then sequenced.

For 3' RACE total RNA was isolated from *Nereis virens *at various stages of development using TRIzol reagent (Invitrogen) according to the manufacturer's instructions. In every case RNA was isolated from about 5000 larvae. Mixed RNA from different larval stages was used as a template for a reverse transcriptase reaction with an oligo-dT-universal primer. Semi-nested PCR was performed using the product of the reverse transcriptase reaction as a template with pairs of gene specific forward primers and a reverse universal primer. Amplified products were cloned in the T Easy vector (Promega) and sequenced. Primers designed using these assembled long sequences were used to generate probes for in situ hybridization by RT-PCR.

### Whole-mount in situ hybridization (WMISH)

Samples were fixed with 4% PFA in 1.75×PBS. A detailed protocol is available upon request. Dig-labeled RNA probes were prepared according to the manufacturer's protocol (Roche). Hybridization was carried out at 68°C. BM-purple (Roche) was used as a chromogenic substrate to localize the hybridized probe [18,48]. The results were imaged on a DMRXA microscope (Leica) with a Leica DC500 digital camera under Nomarsky optics.

## Authors' contributions

MAK and TFA collected *N. virens *at the White Sea. MAK performed all in situ hybridization experiments, made photomicrographs, and prepared figures. TFA cloned fragments of *Nvi-ParaHox *genes, grew larval and postlarval worms, did the regeneration experiments, and wrote the first draft of the manuscript. CEC developed PCR and iPCR protocols, processed and annotated sequences, assembled GenBank submissions, and edited the manuscript. All authors have read and approved the final manuscript.
